# Inhibitory Effects of Naringenin on LPS-Induced Skin Inflammation by NF-κB Regulation in Human Dermal Fibroblasts

**DOI:** 10.3390/cimb46090546

**Published:** 2024-08-23

**Authors:** Yoon-Jung Choy, Gyu-Ri Kim, Hyung-ui Baik

**Affiliations:** 1 Department of Optometry, Eulji University College of Health Sciences, Seongnam 13135, Republic of Korea; yjchoy7@eulji.ac.kr; 2 Department of Bioengineering, Eulji University, Seongnam 13135, Republic of Korea; grkim@eulji.ac.kr; 3 Department of Addiction Rehabilitation and Social Welfare, Eulji University, Seongnam 13135, Republic of Korea

**Keywords:** naringenin, lipopolysaccharide, human dermal fibroblasts, inflammatory agents, cosmetics

## Abstract

Flavonoids are important natural compounds characterized by their extensive biological activities. Citrus flavonoids represent a significant segment of the broader flavonoid category. Naringenin, an integral part of this series, is recognized for its powerful anti-inflammatory and antioxidant properties. In addition, considering the lack of existing research on naringenin’s potential effectiveness and intracellular mechanisms of action in skin-related applications, especially as a cosmetic ingredient, this study aimed to explore naringenin’s role in reducing the fundamental generation of reactive oxygen species. This was achieved by examining its inhibitory effects on the expression levels of NADPH oxidase and iNOS, ultimately leading to a reduction in NO production. This research examined the anti-inflammatory and antioxidant capacities of naringenin by employing a cellular senescence model of LPS-induced HDFs. The evaluation of naringenin’s efficacy was validated through several investigative procedures, including the NF-κB luciferase assay, ELISA assay, and qRT-PCR. To verify the anti-inflammatory effectiveness of naringenin, we measured the responsive elements of NF-κB using a luciferase reporter assay. This assessment revealed that naringenin could decrease the concentration of genes activated by NF-κB. Moreover, we found that naringenin inhibited the transcriptional expression of known NF-κB-regulated inflammatory cytokines, including IL-1β, IL-6, and IL-8. In addition, results from the qRT-PCR analysis indicated that naringenin facilitated a reduction in iNOS expression. Based on the data gathered and analyzed in this study, it can be conclusively inferred that naringenin possesses promising potential as a cosmetic ingredient, offering both anti-inflammatory and antioxidant benefits.

## 1. Introduction

Naringenin, a dominant flavanon in nature, exhibits a broad array of biological and pharmacological activities. The existing body of evidence suggests that naringenin has the ability to modulate acute and chronic inflammatory responses, positioning it as a promising candidate for therapeutic applications. Recent studies have indeed affirmed the effectiveness of naringenin in managing several inflammation-associated diseases, including sepsis, acute hepatitis, fibrosis, and cancer [1,2,3]. Moreover, naringenin has emerged as a significant agent in regulating lipid protein metabolism, offering potential utility in the management of diseases such as diabetes, arteriosclerosis, and insulin resistance, all of which have been extensively covered in previous reviews [4].

Recent research indicates the potential pre- and post-infection effects of naringenin [5]. Similar to other natural compounds, naringenin demonstrates promising outcomes in vitro, but reveals limited results in in vivo viral infection models. Nonetheless, both in vitro and in vivo anti-inflammatory potentials of naringenin have been emphasized, including in various animal models addressing respiratory syndromes. From this standpoint, we highlight the mechanisms through which naringenin could assume a critical anti-inflammatory role in COVID-19.

A variety of substances, including vitamins D, E, and B12, omega-3, and flavonoids, have demonstrated the potential to exhibit antiviral and anti-inflammatory properties, possibly influencing the course of COVID-19. Notably, naringenin (NAR), a crucial natural flavonoid present in citrus fruits, especially in grapefruit (43.5 mg/100 mL) and oranges (2.13 mg/100 mL), showcases analgesic, anti-inflammatory, antitumor, and antiviral effects [6]. It was observed that an intake of 8 mL/kg of orange juice can amplify the concentration of NAR, reaching levels ranging from 0 to 300 µg/L four hours post-consumption [7,8,9,10].

Concerning weight management, naringenin—extracted from several citrus varieties (Sinetrol-XPur), of which 20% is naringenin—has displayed vitality and support for individuals with a BMI in the range of 26 to 29.9 kg/m^2^ [10]. Notable improvements in primary overweight-related endpoints were observed 12 weeks post-initiation, including reductions in waist and hip fat, abdominal fat, and total weight. In addition, there was a marked decrease in both polygon and staging markers [11,12,13]. Stohs and colleagues reported the use of naringin as a supplementary agent (600 mg) in weight management efforts. The lion’s share of recent research trends pertaining to naringrin mainly focus on the fields of medicine, life sciences, and food science, with emphasis on processes, anti-inflammatory actions, and conclusion phases. Nevertheless, studies in the emerging sector are generally minimal, and notably, investigations into the effects of naringenin on human dermal fibroblasts are virtually absent. Therefore, this study intends to showcase the cytoprotective effects of naringenin on ultraviolet ray-affected human dermal fibroblasts, spotlighting the roles of key wrinkle response mediators, such as NFκB activity, COX2, TNFα, IL-6, and IL-8. Through this, this study aimed to investigate the potential of utilizing naringenin as an effective preventive agent in cosmetic formulations.

When cells are treated with LPS, a known initiator of reactive oxygen species (ROS) generation, it triggers a variety of intracellular activation mechanisms, leading to the production of inflammatory cytokines and fetal factors. Inducible nitric oxide synthase (iNOS), a crucial enzyme in generating NO (nitric oxide), functions independently from cellular Ca^2+^ notifications and is predominantly found in macrophages, vascular smooth muscle cells, endothelial cells, and hepatocytes. This enzyme is activated by various stimuli, such as LPS and TNF-α, in a wide array of cells, including myocardial cells, leading to substantial production and secretion of NO. This activation unfortunately gives rise to a plethora of small peroxides and cyclones due to excessive generative activity disruptions, consequently inducing adverse effects stemming from the oxidative destruction of cells [14,15,16,17].

The regulation of the NF-κB system stands as a central factor in addressing the myriad of skin problems common in today’s society, representing ancient signaling pathways that control metabolic processes. This mechanism, governed by opposing regulatory processes, seeks to suppress inflammatory cells in mammals. The primary focus currently lies within the sphere of functional cosmetics and the emerging inner beauty industry, although its efficacy remains somewhat inadequate at present. Hence, we undertook the task of investigating the interplay between naringenin, known for its anti-aging, anti-inflammatory, and antioxidative properties, and the NF-κB pathway.

## 2. Materials and Methods

### 2.1. Cell Culture and Sample Preparation

In this study, human dermal fibroblasts (HDFs) were purchased from Lonza, Visp, Switzerland and cultured in Dulbecco’s Modified Eagle Medium (DMEM; Hyclone, Logan, UT, USA) supplemented with 10% fetal bovine serum (FBS; Hyclone) and 1% penicillin/streptomycin (100 IU/mL penicillin, 100 μg/mL streptomycin; Invitrogen, USA) and cultured at 37 °C and 5% CO_2_ Naringenin (Sigma-Aldrich, St. Louis, MO, USA) was used to dissolve the purified powder (>99%) in a suitable concentration of dimethyl sulfoxide (DMSO; Sigma-Aldrich). Lipopolysaccharide (LPS), a cell-stimulating agent, was purchased from Sigma-Aldrich. For this experiment, the solution was dissolved in dimethyl sulfoxide (DMSO; Sigma-Aldrich) at a proper concentration. HDFs (1 × 106 cells/well) were incubated in a 60 mm cell culture dish for 24 h. An appropriate concentration of naringenin was added to the culture medium. Lipopolysaccharide was co-treated at a constant concentration for 24 h and analyzed 3 h later. The significance of PCR was validated using a melting curve. The gene expressions were compared for analysis by normalizing β-actin expression.

### 2.2. Assessment of Cell Viability

The WST-1 Cell Proliferation Assay System utilizes a mechanism involving the transformation of tetrazolium salts (WST-1) into a color-yielding substance called formazan by mitochondrial dehydrogenase within the cells. This process was employed to quantitatively gauge cell proliferation or survival abilities. During this experimental procedure, HDFs (3 × 10^3^ cells/well) were cultured in a volume of 100 μL per well in a 96-well plate, which facilitated vigorous metabolic activities. Following an incubation period of 24 h, these cells were simultaneously exposed to various agents, including naringenin and LPS, for an additional 24-hour period. After this, 10 μL of the EZ-Cytox cell viability assay kit reagent (ItsBio, Seoul, Republic of Korea) was introduced to the incubated cells and allowed to incubate for an additional hour. The absorbance was then measured at 490 nm using a microplate reader (Bio-Rad, Hercules, CA, USA). This procedure was replicated thrice to calculate the average cell survival rate and the standard deviation.

### 2.3. qRT-PCR Analysis

Changes in gene expression caused by naringenin in the cells were quantitatively confirmed. qRT-PCR was performed by mixing 0.2 μM primers, 50 mM KCl, 20 mM Tris/HCl pH 8.4, 0.8 mM dNTP, 0.5 U Extaq DNA polymerase, 3 mM MgCl 2, and 1X SYBR green (Invitrogen, Carlsbad, CA, USA). The PCR was validated using a melting curve. The melting curve confirmed the validity of PCR. The expression of each gene was standardized and compared. The primers used in this experiment are shown in Table 1.

### 2.4. Enzyme-Linked Immunosorbent Assay (ELISA)

PGE2 (Cayman chemical, Ann Arbor, MI, USA; competitive) was evaluated using an enzyme-linked immunosorbent assay (ELISA) kit according to the instruction manual for the HDF culture medium. Prostaglandin E2 and monoclonal antibodies were immobilized on plastic cell culture dish wells. One hundred microliters of each culture medium was dispensed (at room temperature for 2 h). After washing five times each with a single washing buffer, 100 μL horseradish peroxidase (HRP)-conjugated antibody was added for 1 h at room temperature, and 100 μL of tetramethylbenzidine (TMB) was added and incubated for 30 min in a dark room. The absorbance was measured at 405–420 nm (PGE2).

### 2.5. Statistical Analysis

All experiments were performed independently three times, and the results were expressed as mean ± standard deviation. Student’s t-tests were used to analyze all the findings, with *p* values of below 0.05, 0.01, or 0.001 considered statistically significant (* *p* < 0.05, ** *p* < 0.01, *** *p* < 0.001).

## 3. Results and Discussion

### 3.1. Cytotoxicity Experiments on HDFs

**Figure 1:**
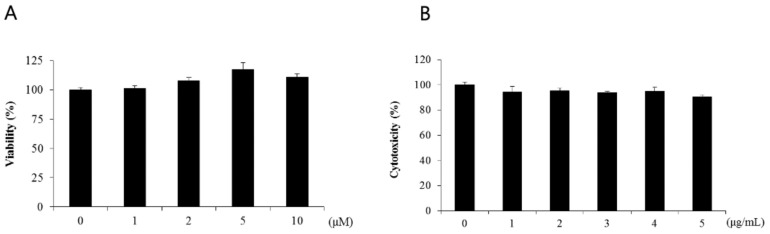
(**A**) The cellular toxicity of naringenin in HDFs. HDFs (1 × 10^6^ cells/well) were cultured in a 60 mm cell culture dish for 24 h. Naringenin was added to the culture medium, and the cells were co-cultured for 24 h at a constant concentration of LPS. The WST-1 assay was used to measure cell viabilities. The results are presented as the averages of three independent experiments, with error bars indicating the standard deviation. (**B**) The cellular toxicity of LPS in HDFs. HDFs (1 × 10^6^ cells/well) were cultured in a 60 mm cell culture dish for 24 h. Naringenin was added to the culture medium, and the cells were co-cultured for 24 h at a constant concentration of LPS. The WST-1 assay was used to measure cell viabilities. The results are presented as the averages of three independent experiments, with error bars indicating the standard deviation.

Lipopolysaccharide acts as an endotoxin in cells, activating signals such as MAPK, NF-κB, and IRF-3 and promoting the excessive secretion of inflammatory cytokines [18]. Lipopolysaccharide induced nuclear factor-кB activity through IκB phosphorylation and its degradation. In this study, WST-1 assay was performed to determine the cytotoxicity of naringenin in human dermal fibroblasts. Human dermal fibroblasts were treated with either an untreated control group or naringenin at 1-, 2-, 5-, and 10-μM dose levels for 24 h. The cell viability of the untreated control group was set at 100%, and up to the 1-μM dose level, the survival rate was shown to be 100% or higher. When naringenin was treated at 1-, 2-, 5-, and 10-μM dose levels, the cell viability levels of 101%, 107%, 117%, and 110% did not decrease, confirming almost no toxicity (Figure 1A). To confirm the changes in the survival rate of the cells, HDFs were treated with LPS at 1-, 2-, 3-, 4-, and 5-μg dose levels and the survival rate was 94% at 1 μg/mL (Figure 1B). The cell viability assay was performed in triplicate and the mean and standard deviation were calculated.

### 3.2. The Impact of Naringenin on HDF Inflammation Suppression Induced by LPS

#### Changes in NF-κB Expression

Within the NF-κB signaling pathway, LPS activates NF-κB through phosphorylation. This activation triggers relocation to the nucleus, stimulating the transcription of inflammatory cytokines and mediators which might induce various diseases [19].

**Figure 2:**
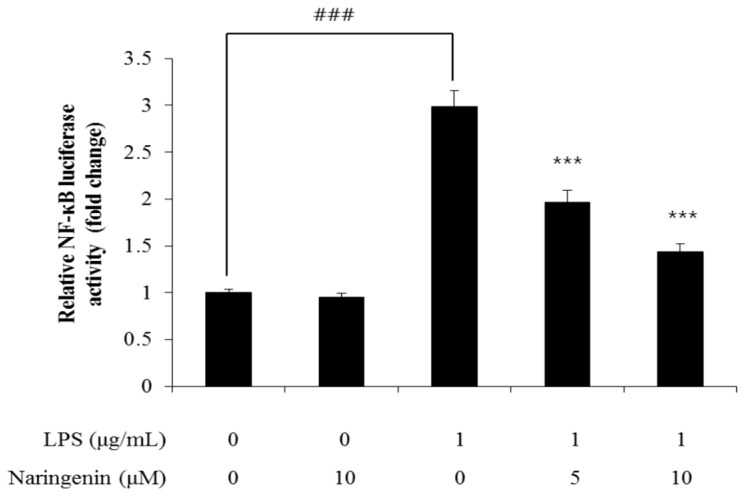
NF-κB reporter NIH-3T3 stable cells were cultured for an additional 24 hours after cellular treatment under optimum conditions. Right after adding luciferin, luminous intensity was measured using a luminometer. The luminous intensity of luciferin decreased in a dose-dependent manner with naringenin. The results are presented as the averages of three independent experiments, with error bars indicating the standard deviation (*** p < 0.001, ### *p* < 0.001).

The NF-κB luciferase assay demonstrated that cellular spacing expanded to a level of 3.0 when treated with LPS. However, subsequent treatment with naringenin at concentrations of 5 and 10 μM resulted in reductions to levels of 2 and 1.4, respectively, illustrating a dose-dependent decrease facilitated by naringenin (Figure 2).

### 3.3. Changes in the Expression of IL-1β, IL-6, and IL-8

The effects of NF-κB activation on HDFs treated simultaneously with LPS and naringenin.

NF-κB reporter NIH-3T3 stable cells were cultured for an additional 24 h after cellular treatment under optimum conditions. Right after adding luciferin, luminous intensity was measured using a luminometer. The luminous intensity of luciferin decreased in a dose-dependent manner with naringenin. The results are presented as the averages of three independent experiments, with error bars indicating the standard deviation (*** *p* < 0.001).

**Figure 3:**
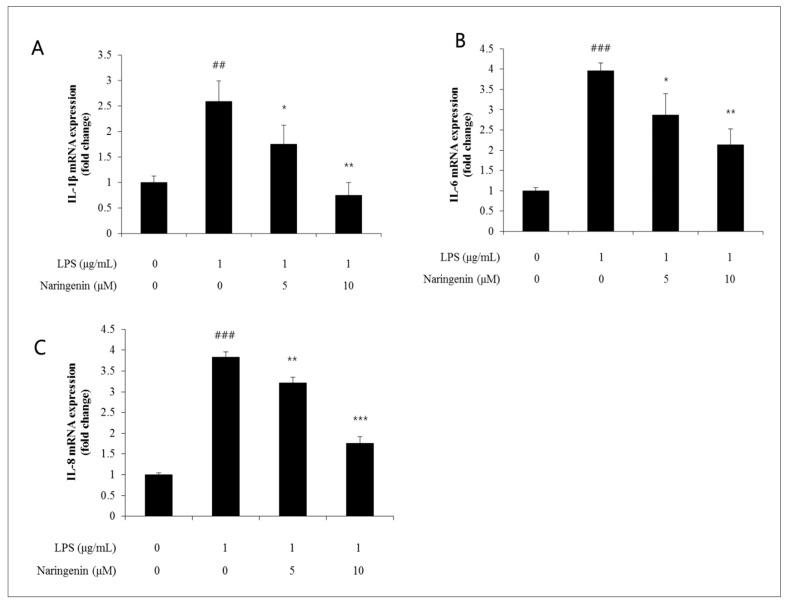
(**A**) IL-1β, a representative cytokine that acts in the early stages of inflammation, increased with LPS but was reduced to 1.8 and 0.8 when treated with naringenin at 5 and 10 μM concentrations, respectively. (**B**) IL-6 which increased with LPS, also decreased to levels of 3.2 and 1.8 when treated with naringenin at 5 and 10 μM concentrations, respectively, and (**C**) IL-8, which increased with LPS, also decreased to levels of 3.2 and 1.8 when treated with naringenin at 5 and 10 μM concentrations, respectively. When treated separately, it decreased to levels of 3.2 and 1.8. (* *p* < 0.05, ** *p* < 0.01, ## *p* < 0.01, *** *p* < 0.001, ### *p* < 0.001).

During the onset of inflammation, IL-1β serves as a significant cytokine that initiates febrile reactions by stimulating various systems, including the hypothalamus–pituitary–adrenal (HPA) axis. This cytokine plays a vital role in activating the immune, neuroendocrine, and neuroimmune systems [20]. Similarly, IL-6 is a cytokine synthesized by a variety of cells, including T lymphocytes, B lymphocytes, neuroglial cells, mast cells, macrophages, endothelial cells, and fibroblasts. It is involved in initiating diverse inflammatory diseases, triggering acute phase reactions, and promoting lymphocyte proliferation. IL-8, another inflammatory cytokine, functions as a chemoattractant for neutrophils, monocytes, and T cells. Belonging to the chemokine superfamily, it is secreted in response to stimuli, such as LPS or leukotrienes, facilitating neutrophil infiltration at the sites of inflammation. Furthermore, IL-8 exerts potent chemoattractive actions on neutrophils, eosinophils, and T lymphocytes, enhancing the expression of adhesion molecules and encouraging the release of lysozyme and reactive oxygen species, thus actively participating in neutrophil activation. Moreover, the release of IL-6 and IL-8 can lead to skin conditions, such as contact dermatitis and atopic dermatitis, potentially intensifying acute responses to various stimuli. This study examined the effects of naringenin on dermal tissues, particularly focusing on fluctuations in the expression levels of the IL-1β gene by utilizing qRT-PCR techniques. When LPS was applied in the presence of naringenin, the intercellular spacing increased to a level of 2.6. This increase was moderated to levels of 1.8 and 0.8 when treated with 5 and 10 μM concentrations of naringenin, respectively, displaying a dose-dependent decline (Figure 3A).

Furthermore, the influence of naringenin on dermal tissues was analyzed by observing alterations in IL-6 gene expression using qRT-PCR methods. After the administration of LPS alongside naringenin, the intercellular space expanded to a level of 3.9, which then decreased to levels of 2.8 and 2.1 with the application of 5 and 10 μM concentrations of naringenin, respectively, indicating a dose-dependent reduction (Figure 3B) In a similar vein, the impact of naringenin on dermal tissues was evaluated by monitoring shifts in IL-8 gene expression using qRT-PCR techniques. Following the introduction of LPS with naringenin, the intercellular distance increased to a level of 3.8, which later contracted to 3.2 and 1.8 upon treatment with 5 and 10 μM concentrations of naringenin, respectively, demonstrating a dose-dependent decrease (Figure 3C).

### 3.4. Changes in the Expression of COX2 and PGE2

The inflammatory mediator, cyclooxygenase (COX), serves as a catalyst in the conversion of arachidonic acid to prostaglandin E2 (PGE2). This enzyme is classified into two subsets: COX1 and COX2. In particular, COX2 is induced within inflammatory cells in response to inflammatory stimuli, and PGE2 generated by COX2 acts as a pivotal inflammatory agent involved in processes such as pain sensation and fever initiation. Moreover, it actively participates in inflammatory responses, immune reactions, and promotes angiogenesis, thus having a profound association with the onset of cancer. PGE2 is recognized as a significant inflammatory molecule, deeply involved in the pathogenesis of various diseases, and is synthesized from arachidonic acid through the action of COX2. This study examined the influence of naringenin on dermal tissue by evaluating changes in COX2 gene expression using qRT-PCR techniques.

**Figure 4:**
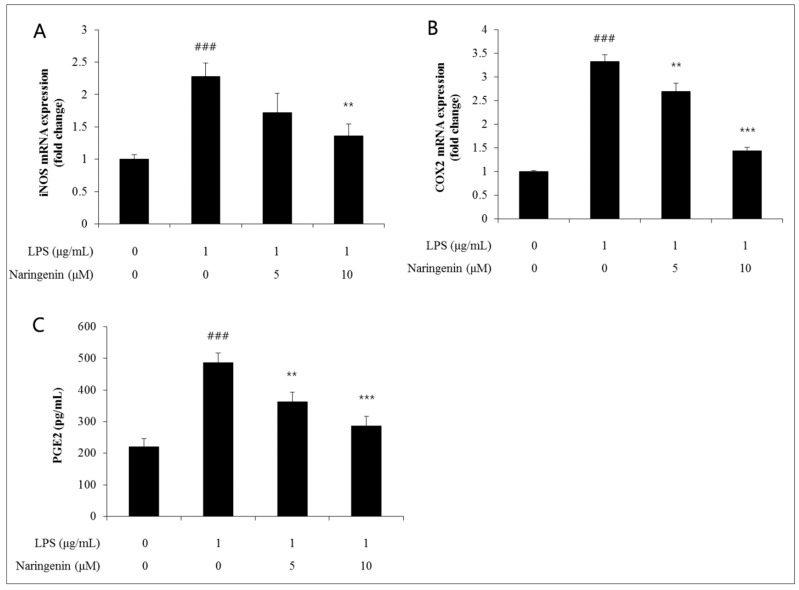
(**A**) In the NADPH oxidase assay, when naringenin was treated at 5 and 10 μM concentrations, it decreased to levels of 2.0 and 1.3, and iNOS increased with LPS and decreased to levels of 1.7 and 1.4 when naringenin was treated at 5 and 10 μM concentrations, respectively. (**B**) COX2 gene expression decreased with naringenin in a dose-dependent manner. (**C**) PGE2 gene expression decreased with naringenin in a dose-dependent manner. The results are presented as the averages of three independent experiments, with error bars indicating the standard deviation (* *p* < 0.05,** *p* < 0.01 *** *p* < 0.001, ### *p* < 0.001).

In the NADPH oxidase assay, treatment with Naringenin at concentrations of 5 and 10 μM resulted in reductions in levels to 2.0 and 1.3, respectively. Furthermore, the levels of iNOS, which were increased by LPS, decreased to 1.7 and 1.4 when treated with Naringenin at the same concentrations. Conversely, the antioxidant enzyme that removes toxic free heme (Fe²+) causing inflammation showed increases to 0.7 and 0.9 upon treatment with 5 and 10 μM Naringenin, respectively (Figure 4A).

We conducted an analysis of the shifts in COX2 gene expression 24 h after treating human dermal fibroblasts (HDFs) with varying concentrations of naringenin, followed by 3-hour LPS treatment. When naringenin was used for the LPS treatment, the intercellular space expanded up to 3.3 μM. This space then contracted to levels of 2.7 and 1.4 when naringenin was applied at concentrations of 5 and 10 μM, respectively, thereby confirming a dose-dependent decrease (Figure 4B). Similarly, the effect of naringenin on dermal tissue was observed by evaluating alterations in PGE2 gene expression using the ELISA assay technique. The cell gap escalated to a level of 487 after LPS treatment with naringenin. This gap reduced to levels of 363 and 287 upon administering naringenin at concentrations of 5 and 10 μM, respectively, thus verifying a decline that was dependent on naringenin concentration (Figure 4C). NF-κB activation effects on HDFs treated simultaneously with LPS and naringenin.

NF-κB reporter NIH-3T3 stable cells were cultured for an additional 24 h after cellular treatment under optimum conditions. Right after adding luciferin, luminous intensity was measured using a luminometer. The luminous intensity of luciferin decreased in a dose-dependent manner with naringenin. The results are presented as the averages of three independent experiments, with error bars indicating the standard deviation (*** *p* < 0.001).

## 4. Discussion

In recent times, the escalation of air pollution, particularly from particulate matter, has been identified as a significant contributor to the onset of various ailments, including respiratory diseases. This issue is exacerbated in our locale by the supplementary pollutants emitted from factories and automobiles, which have a detrimental effect on the health of our community. It is widely acknowledged that the conventional aging process is dictated by genetic determinants and unfolds gradually over time, a phenomenon referred to as intrinsic skin aging. Accordingly, it is evident that the recent influx of particulate matter originating from China not only exacerbates atopic dermatitis and accelerates skin aging, but also increases the likelihood of heightened sensations of skin irritation and itchiness, particularly among individuals with sensitive skin.

When it comes to treating dermatitis, steroids are commonly prescribed. However, their protracted use can lead to adverse side effects, such as skin atrophy and vascular dilation. Recently, there has been a surge in interest in nanoparticle-based therapies for atopic dermatitis, with a particular focus on essential oils. These oils are notable for their small molecular size, which facilitates easy absorption, and they possess moisturizing, anti-inflammatory, and antibacterial properties, thereby intensifying research efforts to explore their efficacy in alleviating atopic dermatitis [18,19,20].

Extensive research has established that NF-κB, a transcription factor responsive to redox changes, plays a pivotal role in regulating the gene expression of various pro-inflammatory cytokines that drive the inflammatory response associated with aging [20]. This study investigated the effects of naringenin on cellular aging suppression and its anti-inflammatory properties by utilizing several aging-related biomarkers. This study further substantiated naringenin’s inhibitory influence on NF-κB activation, a central mediator in inflammatory responses, by analyzing the expression of genes that are upregulated in response to NF-κB activation. After employing ELISA assays and qRT-PCR, we observed a dose-dependent reduction in the levels of PGE2, IL-6, and IL-1β. Moreover, NF-κB activation was quantified using a luciferase assay, confirming that naringenin effectively attenuates NF-κB activity in a concentration-dependent manner. These results demonstrate that naringenin inhibits inflammation in vitro by downregulating the gene expression of IL-1β, TNF-α, and COX2—key constituents of the senescence-associated secretory phenotype (SASP)—by blocking the NF-κB signaling pathway. Thus, naringenin emerges as a promising novel cosmetic ingredient, offering antioxidative properties, anti-inflammatory effects, and protective benefits against skin aging. This study further underscores the critical role of NF-κB activation in both initiating and exacerbating the aging process by examining the relationship between the secretion of proteins associated with aging-related secretory phenotypes (SASPs) and the aging mechanism itself [21,22,23,24,25]. Consequently, this research delved into the gene expression of inflammatory mediators, which are fundamental components of SASP, including NF-κB-regulated genes such as IL-1β, IL-6, IL-8, TNF-α, COX2, and PGE2.

## 5. Conclusions

By inhibiting the gene expression of these primary SASP components by blocking the NF-κB pathway using naringenin, this study aimed to ascertain its potential as an effective anti-inflammatory agent in vitro, setting the stage for further investigations into its prospects as an ingredient in anti-aging cosmetic products.

Firstly, we conducted an extensive analysis of the impact of naringenin on cellular aging. Treatment and psychological suppression alleviation was achieved by using biomarkers for a thorough investigation. During LPS treatment, as depicted in the NF-κB luciferase analysis, the intercellular gap expanded to a level of 3.0. However, this gap reduced to levels of 2.0 and 1.4 when treated with naringenin concentrations of 5 and 10 μM, respectively. Significantly, the cytokine IL-1β, which initially increased due to LPS, exhibited reduced levels of 1.8 and 0.8 when administered with naringenin at 5 and 10 μM concentrations, respectively. Concurrently, IL-6 and IL-8 levels, which increased due to LPS, were recorded at levels of 2.8 and 2.1, and were expected to decreased to levels of 3.2 and 1.8, respectively, when handled with the same naringenin concentrations. Meanwhile, the LPS-induced surge in COX2 was noted at levels of 2.7 and 1.4 upon administering 5 and 10 μM of naringenin, respectively. Furthermore, PGE2 levels were notably high, registering levels of 363 and 287 under similar conditions.

Secondly, we noted the antioxidative suppression effects of naringenin and NAC in inhibiting the formation of antimicrobial oxidative substances triggered by LPS. Aside from the untreated cases, which maintained a 100% rate, the total ROS quantity increased to a level of 2.1 following LPS treatment. Conversely, levels of 1.7 and 1.2 were documented when naringenin was applied at concentrations of 5 and 10 μM. It was expected to stabilize at a level of 1.0 upon the administration of 10 mM of NAC. Experimental data suggest that at a lower concentration of 10 μM, naringenin enhances the ability of skin cells to disperse ROS more effectively than the control group treated with 10 mM of NAC, thus promoting an antioxidative suppression effect. The NADPH oxidase analysis demonstrated levels of 2.0 and 1.3 when managed with naringenin at 5 and 10 μM, and the increase in iNOS due to LPS manifested at levels of 1.7 and 1.4 under similar circumstances. In opposition to the established views regarding LPS and the removal of free iron (Fe^2+^), this analysis revealed maintenance levels of 0.7 and 0.9 when treated with naringenin at concentrations of 5 and 10 μM.

The results of this study suggest that naringenin can be used as a skin anti-aging ingredient. It inhibits various cytokines and ROS-generating substances by regulating the activity of NF-ҡB, which is a major contributor to modern skin aging, and can be used to treat problematic skin diseases.

## Figures and Tables

**Table 1:** Lists of primers.

Gene	Forward Primer	Forward Primer
*IL-6*	TAACAGTTCCTGCATGGGCGGC	AGGACAGGCACAAACACGCACC
*IL-8*	CTCTCTTGGCAGCCTTCCCTC	AATCACTCTCAGTTCTTTG
*IL-1β*	GATCCACACTCTCCAGCTGCA	CAACCAACAAGTGATATTCTCCATG
*COX2*	CGCGGATCCGCGGTGAGAACCGTTTAC	GCGAGGAAGCGGAAGAGTCTAGAGTCGACC
*iNOS*	GCGTTACTCCACCAACAATGGCAA	ATAGAGGATGAGCTGAGCATTCCA

## Data Availability

The data presented in the study are included in this article, further inquiries can be directed to the corresponding author.
